# On the Use of Self-Organizing Map for Text Clustering in Engineering Change Process Analysis: A Case Study

**DOI:** 10.1155/2016/5139574

**Published:** 2016-12-04

**Authors:** Massimo Pacella, Antonio Grieco, Marzia Blaco

**Affiliations:** Dipartimento di Ingegneria dell'Innovazione, Università del Salento, 73100 Lecce, Italy

## Abstract

In modern industry, the development of complex products involves engineering changes that frequently require redesigning or altering the products or their components. In an engineering change process, engineering change requests (ECRs) are documents (forms) with parts written in natural language describing a suggested enhancement or a problem with a product or a component. ECRs initiate the change process and promote discussions within an organization to help to determine the impact of a change and the best possible solution. Although ECRs can contain important details, that is, recurring problems or examples of good practice repeated across a number of projects, they are often stored but not consulted, missing important opportunities to learn from previous projects. This paper explores the use of Self-Organizing Map (SOM) to the problem of unsupervised clustering of ECR texts. A case study is presented in which ECRs collected during the engineering change process of a railways industry are analyzed. The results show that SOM text clustering has a good potential to improve overall knowledge reuse and exploitation.

## 1. Introduction

The development of complex products, such as trains or automobiles, involves engineering changes that frequently require redesigning or altering the products and their components. As defined by Jarratt et al. [1] “engineering change is an alteration made to parts, drawings or software that have already been released during the design process. The change can be of any size or type, can involve any number of people and can take any length of time.” A change may encompass any modification to the form, fit, and/or function of the product as a whole or in part, materials, and may alter the interactions and dependencies of the constituent elements of the product. A change can be needed to solve quality problems or to meet new customer requirements. Although engineering change management was historically seen as a typical design and manufacturing research field, several contributions highlighted the effect of engineering change on other business processes such as material requirement planning [2] and enterprise resource planning [3, 4]. An overview of the engineering change process and a big picture of literature on engineering change management are provided, respectively, by Jarratt et al. [5] and Hamraz et al. [6].

The engineering change request (ECR) is the document which initiates the engineering change process. ECR is used to describe a required change or a problem which may exist in a given product. After the ECR, the impact of a change is discussed among involved stakeholders and the best possible solution is identified.

Once the implementation of a change is completed, too often ECRs are no longer consulted by who could benefit from them. However, reviewing the ECR documents could offer a chance to improve both the design of a product and the engineering change process. A change may be a chance to both improve the product and do things “better next time” [9]. ECRs are documents containing structured and unstructured data, which, if analyzed, may be useful to discover information relating to recurring problems and solutions adopted in the past.

As described in Hamraz et al. [6], a lot of literature concerns the prechange stage of the process and proposes methods to prevent or to ease the implementation of engineering changes before they occur. In contrast, the postchange stage involves less publication and deals with the ex post facto exploration of effect of implemented engineering changes. The analysis of engineering changes process belongs to the postchange stage and there are only few approaches concerning the analysis of engineering changes data in complex products industry. In this context, one of the main challenges is dealing with free-form text contained in engineering changes documents which makes the data more difficult to query, search, and extract. This paper focuses on unstructured data contained in ECRs and proposes the text clustering for the postchange analysis of engineering change process.

Text clustering is an unsupervised learning method where similar documents are grouped into clusters. The goal is to create clusters that are coherent internally, but clearly different from each other. Among the clustering methods proposed in the literature, Self-Organizing Map (SOM) has attracted many researchers in recent years. SOM is a neural-network model and algorithm that implements a characteristic nonlinear projection from the high-dimensional space of input signals onto a low-dimensional regular grid, which can be effectively utilized to visualize and explore properties of the data [10]. With respect to other text clustering methods, SOM allows visualizing the similarity between documents within the low-dimensional grid. Hence, similar documents may be found in neighboring regions of the grid.

In the literature, text mining methods have been proposed in support of the engineering change process by Sharafi et al. [11], Elezi et al. [12], and Sharafi [13]. In particular, Sharafi et al. [11] focused on the causes of changes contained in ECRs and calculated term occurrences for all ECRs in order to analyze occurrences of the keywords in different projects and to find pattern in the data. Elezi et al. [12] employed a semiautomatic text mining process to classify the causes of iteration in engineering changes. As a result, cost and technical categories of causes were identified as the main reasons for the occurrence of iterations. Sharafi [13] applied Knowledge Discovery in Database methods to analyze historical engineering changes data in order to gain insights in form of patterns within the database. In detail, a part of the study concerned the application of *K*-means, *K*-Medoids, DBSCAN, and Support Vector Clustering methods to cluster ECRs documents of an automotive manufacturer.

This paper explores the use of SOM to the problem of unsupervised clustering of ECR documents. A case study is presented and ECRs collected during the engineering change process of a railway industry are analyzed. The results show that SOM text clustering has great potential to improve overall knowledge reuse and exploitation in an engineering change process.

The reminder of the paper is organized as follows. In Section 2, the basic concepts of the SOM theory are introduced. In Section 3, the SOM text based clustering method is described. In Section 4, the engineering change process in industry is described. In Section 5, the case study and the experimental results are both discussed. In Section 6, conclusions are given.

## 2. The SOM Algorithm

The SOM, originally proposed by Kohonen [14], is based on the idea that systems can be designed to emulate the collective cooperation of the neurons in the human brain. It is an unsupervised machine learning method widely used in data mining, visualization of complex data, image processing, speech recognition, process control, diagnostics in industry and medicine, and natural language processing [15].

**Figure 1:**
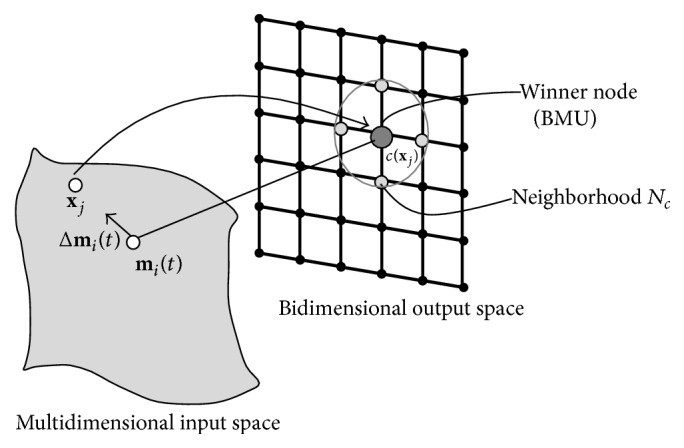
SOM training algorithm. Each neuron has a prototype vector **m**_*i*_, which corresponds to a point in the embedding input space. An input vector **x**_*j*_ will select that neuron with closest **m**_*i*_ to it. Adjustment of the weight vector for the winning output neuron and its neighbors through quantity Δ**m**_*i*_. Adapted from Ritter et al. [7].

The algorithm of SOM consists in mapping *M*-dimensional input vectors **x**_*j*_ to two-dimensional *neurons* or *maps* according to their characteristic features. It reduces the dimensions of data to a map, helps to understand high-dimensional data, and groups similar data together. A simple SOM consists of two layers. The first includes nodes in the input space and the second the nodes in the output space. A representation of SOM with output nodes in a two-dimensional grid view is provided in Figure 1. SOM consists of *P* units; each unit of index *i* is associated with an *M*-dimensional prototype vector **m**_*i*_ in the input space and a position vector on a low-dimensional regular grid, **r**_*i*_, in the output space. The steps of the SOM learning algorithm are as follows:

(1) *Initialization.* Start with initial values of prototype vectors **m**_*i*_. In the absence of any prior information, values of prototype vector **m**_*i*_ can be random or linear and are adjusted while the network learns.
(2) *Sampling.* Select a vector **x**_*j*_ from the training input space. The selection of **x**_*j*_ can be random.
(3) *Matching.* Determine the Best Matching Unit (BMU). Vector **x**_*j*_ is compared with all the prototype vectors and the index *c*(**x**_*j*_) of the BMU; that is, the prototype vector **m**_*c*_ which is closest to **x**_*j*_ is chosen accordingly to the smallest Euclidian distance as follows:
  (1) $$\begin{array}{c} \left\|\;x_{j}-m_{c}\;\right\|=\underset{i}{\min}\left\|\;x_{j}-m_{i}\;\right\|. \end{array}$$
(4) *Updating.* Update the BMU and its neighbors. Adjustment of the prototype vector for the winning output neuron and its neighbors are updated as
  (2) $$\begin{array}{c} m_{i}\left(\;t+1\;\right)=m_{i}\left(\;t\;\right)+\Delta m_{i}\left(\;t\;\right), \end{array}$$
 where *t* = 0,1, 2,… is an index of the time. The value of Δ**m**_*i*_(*t*) in (2) is computed as follows:
  (3) $$\begin{array}{c} \Delta m_{i}\left(\;t\;\right)=\alpha \left(\;t\;\right)h_{ci}\left(\;t\;\right)\left(\;x_{j}\left(\;t\;\right)-m_{i}\left(\;t\;\right)\;\right), \end{array}$$
 where *α*(*t*) is the learning-rate factor and *h*_*ci*_(*t*) the neighborhood function. In particular, the learning-rate factor *α*(*t*) is comprised in [0,1] and is monotonically decreasing during the learning phase. The neighborhood function *h*_*ci*_(*t*) determines the distance between nodes of indexes *c* and *i* in the output layer grid. A widely applied neighborhood kernel can be written in terms of the Gaussian function:
  (4) $$\begin{array}{c} h_{ci}\left(\;t\;\right)=\exp\left(\;-\frac{{\left\|r_{c}-r_{i}\right\|}^{2}}{2\sigma^{2}\left(t\right)}\;\right), \end{array}$$
 where **r**_*c*_ and **r**_*i*_ are the position vectors of nodes *c* and *i* and the parameter *σ*(*t*) defines the width of the kernel which corresponds to the radius of the neighborhood *N*_*c*_(*t*). *N*_*c*_(*t*) refers to a neighborhood set of array points around node of index *c* (Figure 1). The value *h*_*ci*_(*t*) decreases during learning, from an initial value often comparable to the dimension of the output layer grid to a value equal to one.

During the learning of the SOM, phases 2–4 are repeated for a number of successive iterations until the prototype vectors **m**_*i*_ represent, as much as possible, the input patterns **x**_*j*_ that are closer to the neurons in the two-dimensional map. After initialization, the SOM can be trained in a sequential or batch manner [8]. Sequential training is repetitive as batch training but instead of sending all data vectors to the map for weight adjustment, one data vector at a time is sent to the network. Once the SOM is trained, each input vector is mapped to one neuron of the map, reducing high-dimensional input space to a low-dimensional output space. The map size depends on the type of application. The bigger size map reveals more details of information whereas a smaller map is being chosen to guarantee the generalization capability.

Before application, SOM method requires predefining the size and structure of the network, the neighborhood function, and the learning function. These parameters are generally selected on the basis of heuristic information [7, 8, 16, 17].

### 2.1. SOM Cluster Visualization

The SOM is an extremely versatile tool for visualizing high-dimensional data in low dimension. For visualization of SOM both the unified-distance matrix (U-matrix) [18] and Component Planes [19] are used. The U-matrix calculates distances between neighboring map units, and these distances can be visualized to represent clusters using a color scale on the map.

The U-matrix technique is a single plot that shows cluster borders according to dissimilarities between neighboring units. The distance ranges of U-matrix visualized on the map are represented by different colors (or grey shades). Red colors correspond to large distances; that is, large gaps exist between the prototype vector values in the input space; blue colors correspond to small distance; that is, map units are strongly clustered together. U-matrices are useful tools for visualizing clusters in input data without having any a priori information about the clusters.

Another important tool of visualization is Component Planes, that is, a grid whose cells contain the value of the *p*th dimension of a prototype vector displayed by variation of color. It helps to analyze the contribution of each variable to cluster structures and the correlation between the different variables in the dataset.

### 2.2. SOM Clustering Using *K*-Means Algorithm

One of the drawbacks of SOM analysis is that unlike other cluster methods, the SOM has no distinct cluster boundaries. When datasets become more complex it is not easy to distinguish the cluster by pure visualization. As described in Vesanto and Alhoniemi [10], in SOM the prototype nodes can be used for clustering instead of all input dataset. Let *𝒞*_1_,…, *𝒞*_*k*_,…, *𝒞*_*K*_ denote a cluster partition composed of *K* clusters. The choice of the best clustering can be determined by applying the well-known *K*-means algorithm [20]. This algorithm minimizes an error function computed on the sum of squared distances of each data point in each cluster. The algorithm iteratively computes partitioning for the data and updates cluster centers based on the error function. In this approach, the number of clusters *K* has to be fixed a priori. Therefore *K*-means algorithm is run multiple times for each $K\in [2,\sqrt{N}]$, where *N* is number of samples. The best number of clusters *K* ^*∗*^ can be selected based on the Davies Bouldin Index (DBI) [21]. This index is based on a ratio of within-cluster and between-cluster distances and is calculated as

(5) $$\begin{array}{c} DBI\left(\;K\;\right)=\frac{1}{K}\overset{K}{\underset{k=1}{\sum }}\underset{k\neq l}{\max}\left\{\;\frac{\Delta \left(C_{k}\right)+\Delta \left(C_{l}\right)}{\delta \left(C_{k},C_{l}\right)}\;\right\}, \end{array}$$

where *K* is the number of clusters, Δ(*𝒞*_*k*_) and Δ(*𝒞*_*l*_), and *δ*(*𝒞*_*k*_, *𝒞*_*l*_) the within-cluster and between-cluster distances, respectively. The optimum number of clusters *K* ^*∗*^ corresponds to the minimum value of DBI(*K*). SOM neural network combined with other clustering algorithms was used in Yorek et al. [22] for visualization of students' cognitive structural models.

## 3. SOM-Based Text Clustering

Text clustering is an unsupervised process used to separate a document collection into some clusters on the basis of the similarity relationship between documents in the collection [17]. Suppose *𝒞* = {*d*_1_,…, *d*_*j*_,…, *d*_*N*_} be a collection of *N* documents to be clustered. The purpose of text clustering is to divide *𝒞* into *𝒞*_1_,…, *𝒞*_*k*_,…, *𝒞*_*K*_ clusters, with *𝒞*_1_ ⋯ ∪*𝒞*_*k*_ ⋯ ∪*𝒞*_*K*_ = *𝒞*.

SOM text clustering can be divided into two main phases [23, 24]. The first phase is* document preprocessing* which consists in using Vector Space Model (VSM) to generate output document vectors from input text documents. The second one is* document clustering* that applies SOM on the generated document vectors to obtain output clusters.

### 3.1. Document Preprocessing

An important preprocessing aspect for text clustering is to consider how the text content can be represented in the form of mathematical expression for further analysis and processing.

By means of VSM, each *d*_*j*_ (*j* = 1,…, *N*) can be represented as vector in *M*-dimensional space. In detail, each document *d*_*j*_ can be represented by a numerical feature vector **x**_*j*_:

(6) $$\begin{array}{c} x_{j}=\left[\;w_{1,j},\ldots ,w_{p,j},\ldots ,w_{M,j}\;\right]. \end{array}$$

Each element *w*_*p*,*j*_ of the vector usually represents a word (or a group of words) of the document collection; that is, the size of the vector is defined by the number of words (or groups of words) of the complete document collection.

The simplest approach is to assign to each document the* Term Frequency and Inverse Document Frequency* (TF-IDF) weighting scheme [25, 26]. The TF-IDF weighting scheme assigns to each term *p* in the *j*th document a weight *w*_*p*,*j*_ computed as

(7) $$\begin{array}{c} w_{p,j}=tf_{p,j}\times \log\frac{N}{df_{p}}, \end{array}$$

where *tf*_*p*,*j*_ is the term frequency; that is, the number of times that term *p* appears in the document *j* and *df*_*p*_ is the number of documents in the collection which contains term *p*.

According to TF-IDF weighting scheme, *w*_*p*,*j*_ is

(1) higher when the term *p* occurs many times within a small number of documents (thus lending high discriminating power to those documents),
(2) lower when the term *p* occurs fewer times in a document or occurs in many documents (thus offering a less pronounced relevance signal),
(3) lower when the term *p* occurs in virtually all documents.

Before preprocessing the documents by the TF-IDF weighting scheme, The size of the list of terms created from documents can be reduced using methods of* stop words removal* and* stemming* [23, 24].

In text based document, in fact, there are a great number of noninformative words, such as articles, prepositions, and conjunctions, called stop words. A stop-list is usually built with words that should be filtered in the document representation process. Words that are to be included in the stop-list are language and task dependent; however a set of general words can be considered stop words for almost all tasks, such as “and” and “or.” Words that appear in very few documents are also filtered.

Another common phase in preprocessing is stemming, where the word stem is derived from the occurrence of a word by removing case and inflection information. For example, “computes,” “computing,” and “computer” are all mapped to the same stem “comput.” Stemming does not alter significantly the information included in document representation, but it does avoid feature expansion.

### 3.2. SOM Text Clustering

Once obtaining the feature vector **x**_*j*_ in (6) associated with each text *d*_*j*_, the SOM algorithm described in Section 2 can be applied for text clustering. The text clustering method explained above is known as “SOM plus VSM”; other variants to it have been proposed by Liu et al. [27, 28]. An overview of the application of SOM in text clustering is provided by Liu et al. [17]. This kind of clustering method was employed in domains such as patent [29], financial services [30], and public policy analysis [31].

## 4. The Engineering Change Process in Complex Products Industry

For complex products, such as trains, automobiles, or aircraft, engineering changes are unavoidable and products or components have to be redesigned and retrofitted to accommodate the new changes to new installations and products. In these environments, an engineering change can involve the risk of due time delay. Huang et al. [32] carried out a survey about the effects of engineering changes on four manufacturing industries and found that the time invested in processing an engineering change varies from 2 to 36 person days. In Angers [33] it is estimated that more than 35% of today's manufacturing resources are just devoted to managing changes to engineering drawings, manufacturing plans, and scheduling requirements. Engineering change processes in complex environments such as automobile, train, and aviation industry were also studied by Leng et al. [3] and Subrahmanian et al. [34].

**Figure 2:**
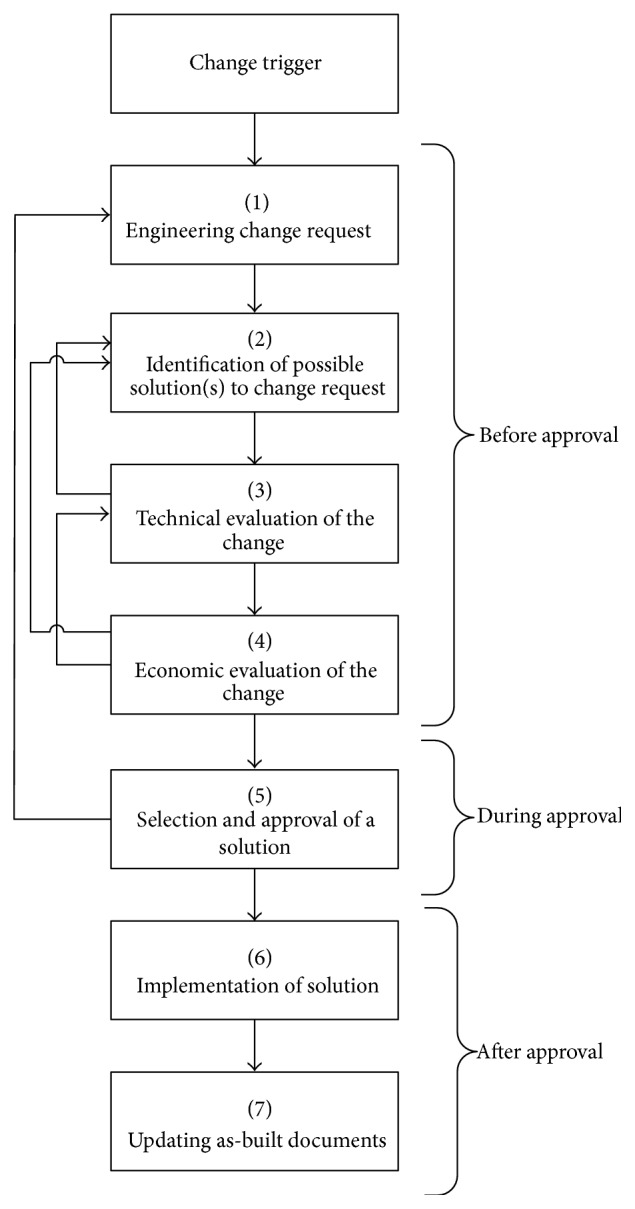
The real engineering change process in complex products environments. Adapted from Jarratt et al. [5].

The phases of a real engineering change process in complex products industry can be summarized as follows (Figure 2):

1. A request of an engineering change is made and sent to an engineering manager. In this phase, standard ECR forms are used outlining the reason of the change, the type of the change, which components or systems are likely to be affected, the person and the department making the request, and so forth.
2. Potential solutions to the request for change are identified.
3. Technical evaluation of the change is carried out. In this phase, the technical impact of implementing each solution is assessed. Various factors are considered, for example, the impact upon design and product requirements, production schedule, and resources to be devoted.
4. Economic evaluation of the change is performed. The economic risk of implementing each solution is assessed. In this phase, costs related to extra production times, replacements of materials, penalty for missed due date, and so forth are estimated.
5. Once a particular solution is selected, it is approved or not approved. The change is reviewed and a cost benefit analysis is carried out. When a solution is approved, the engineering change order is prepared and issued.
6. Implementation of the engineering change and identification of the documents, such as drawings, are to be updated.
7. Update of the as-built documents occurs. As-built documents are usually the original design documents revised to reflect any changes made during the process, that is, design changes, material changes, and so forth.

Iterations of the process occur, for example, when a particular solution has negative impact on product requirements or is too risky to be implemented so the process returns to phase 2 and another solution is identified. Another iteration is possible when the costs of a solution are too high or more risk analysis is required or when the proposed solution is completely refused.

As shown in Figure 2, no review process of similar changes faced in the past is carried out during the process or at the end. This aspect is emphasized by Jarratt et al. [5] by highlighting that, after a period of time, the change should be reviewed to verify if it achieved what was initially intended and what lessons can be learned for future change process. Various factors can discourage examining the solutions adopted in the past to a particular change. First of all, there is the lack of opportune methods to analyze the documents collected during the process, that is, ECR. ECRs are often forms containing parts written in natural language. Analyzing these kinds of documents in the design phase of a product or a component or when a new change request occurs could be very time consuming without an appropriate solution.

In this context, SOM text clustering application can improve the process. When a new ECR occurs, in fact, ECRs managed in the past and similar to the current request could be analyzed in order to evaluate the best solution and to avoid repeating the same mistakes made in the past. In order to explore the existence of similarity between the different ECRs texts, the first step is to verify the potential clustering present in the analyzed dataset. The application of SOM text clustering to ECR documents is explored in the next section.

## 5. The Use of SOM Text Clustering for ECRs Analysis

In order to test SOM text clustering, we used a dataset of *N* = 54 ECRs representing some engineering changes managed during the engineering change process of a railway company. The dataset included the natural language written descriptions of the causes of changes contained in the ECRs forms. The documents were written in Italian language and the VSM in Section 3 was used to generate output vectors from input text documents. The number of terms, that is, the dimension of each vector **x**_*j*_ associated with each document in the dataset after the* stop word removal* and* stemming* processes, was equal to *M* = 361.

In our work, we used the MATLAB software. Specifically, Term to Matrix Generator (TMG) toolbox [35] for document preprocessing and SOM toolbox for SOM text clustering [8] were employed.

The map size of the SOM is computed through the heuristic formula in Vesanto et al. [8]. In detail, the number of neurons is computed as $5\sqrt{N}$, where *N* is the number of training samples. Map shape is a rectangular grid with hexagonal lattice. The neighborhood function is Gaussian and the map is trained using the batch version of SOM. After the map is trained, each data vector is mapped to the most similar prototype vector in the map, that is, the BMU which results from the matching step in the SOM algorithm. In our case, the network structure is a 2D-lattice of 7 × 5 hexagonal.

A first step in cluster analysis based on SOM is based on visual inspection through U-matrix and Component Planes.

**Figure 3:**
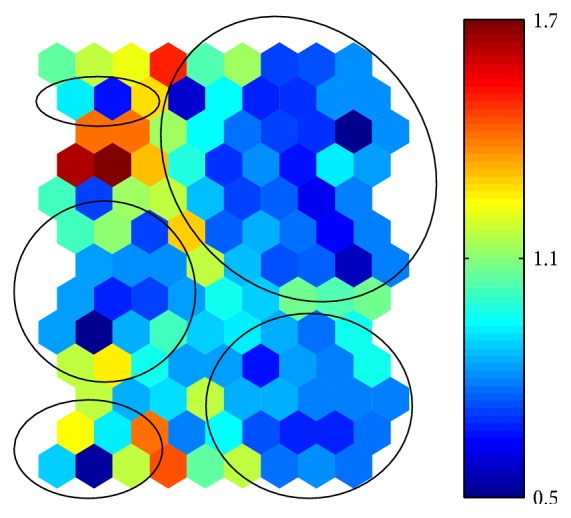
U-matrix of the 54 ERCs texts preprocessed through the VSM. Color scale is related to distances between map units. Red colors represent large distances and blue colors represent small distances.

The U-matrix obtained on the application of SOM to ECR dataset is shown in Figure 3. In order to represent additional information (i.e., distances), the SOM map size is augmented by inserting an additional neuron between each pair of neurons and reporting the distance between the two neurons. The U-matrix and the different colors linked to the distance between neurons on the map show that five clusters are present in the dataset. Each cluster has been highlighted by a circle.

**Figure 4:**
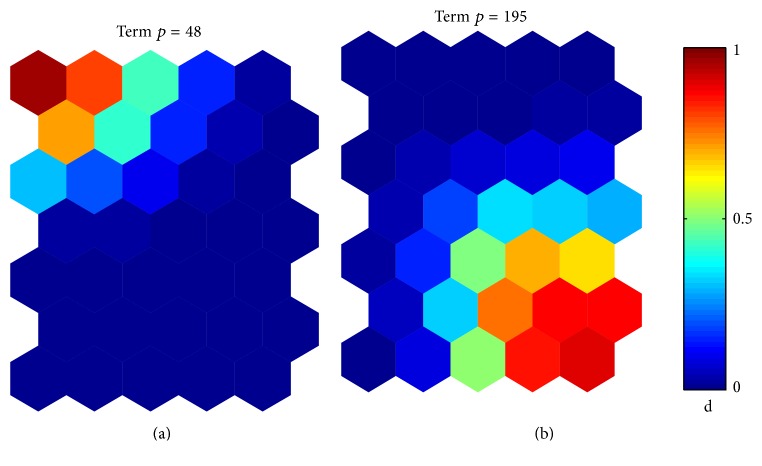
Component Planes of two variables chosen as example. (a) Component Plane related to the variable of index *p* = 48 corresponding to the term “Antenna.” (b) Component Plane of the variable of index *p* = 195 corresponding to the term “Metal-Sheet.” (a) and (b) are linked by position: in each figure, the hexagon in a certain position corresponds to the same map units. Each Component Plane shows the values of the variable in each map unit using color-coding. Letter “d” means that denormalized values are used; that is, the values in the original data context are used [8].

Besides looking at the overall differences in the U-matrix, it is interesting as well to look at the differences between each component present in the input vectors, meaning that we look at differences regarding each component associated with a single “term” in the input dataset. The total number of Component Planes obtained from the SOM corresponds to the total number of terms in our dataset; that is, *M* = 361. For illustrative purpose, Figure 4 shows two Component Planes chosen as an example. The first Component Plane (Figure 4(a)) is associated with the term of index *p* = 48, that is, the term “Antenna”; the second one (Figure 4(b)) is related to the term of index *p* = 195, that is, the term “Metal-Sheet.” The difference between the two terms in the dataset can be represented by considering, for example, the map unit in top left corner of the two figures. This map unit has high values for variable “Term *p* = 48” and low values for variable “Term *p* = 195.” From the observation of Component Planes, we can conclude that there is no correlation between the two terms. As a matter of fact, these two terms were never used together into the same documents.

**Figure 5:**
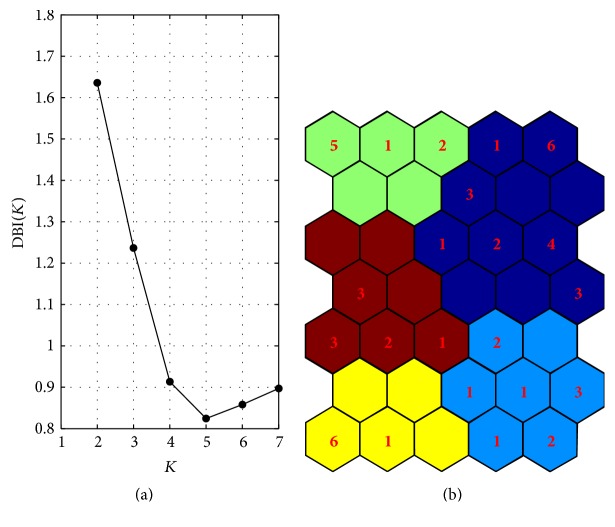
(a) Davies Bouldin Index (DBI) values for number of clusters *K* in [2,7]. (b) Clustering result of SOM using *K*-means with *K* ^*∗*^ = 5 clusters. In each hexagon the number of documents sharing the same BMU is provided.

As shown above, the U-matrix and the Component Planes allow obtaining a rough cluster structure of the ECR dataset. To get a finer clustering result, the prototype vectors from the map were clustered using *K*-means algorithm. The best number of clusters in the SOM map grip can be determined by using the DBI values.  Figure 5(a) shows the DBI values by varying the number of clusters *K* in [2,7]. The elbow point in the graph shows that the optimum number of cluster *K* ^*∗*^ corresponding to the minimum value of DBI(*K*) is equal to 5. The clustering result of SOM obtained by using *K*-means with *K* ^*∗*^ = 5 clusters is shown in Figure 5(b). The BMUs belonging to each cluster are represented with a different color and in each hexagon the number of documents associated with each BMU is provided.

### 5.1. External Validation of SOM Text Clustering

In the reference case study, the true classification of each ECR in the dataset was provided by process operators. Therefore, this information was used in our study in order to perform an external validation of the SOM text clustering. In particular, each ECR text was analyzed and classified with reference to the main component involved in the engineering change (namely, “Metal-Sheet,” “Carter,” “Antenna,” “Semi-Finished Round,” “Hydraulic Panel,” and “Pneumatic System”).  Table 1 reports the number of ECRs related to each component, along with the labels used in order to classify the ECR documents (namely, “MS,” “CR,” “AN,” “SR,” “HP,” and “PS,” respectively). Although a classification is available in the specific case study of this paper, it is worth noting that often such information may be unavailable.

**Figure 6:**
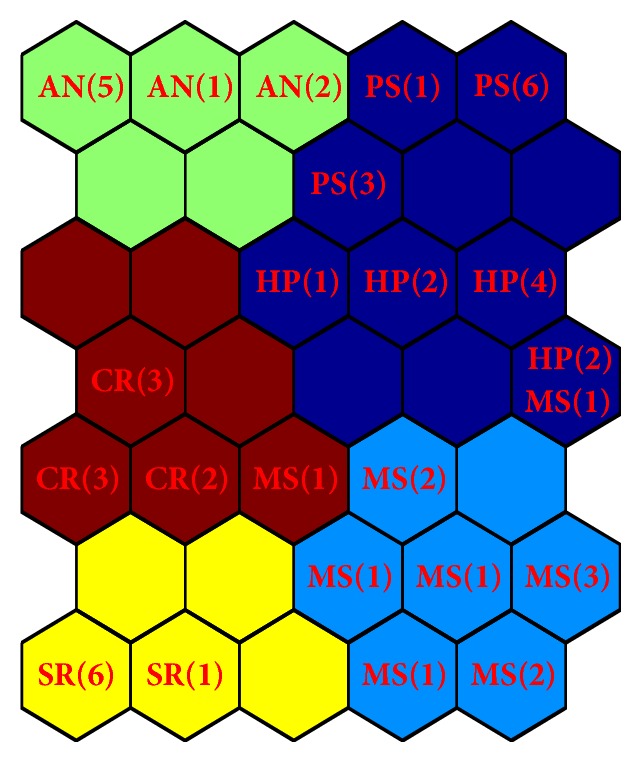
Labeled map grid of SOM. In each hexagon, labels and total number of documents sharing the same BMU. Coloring refers to *K*-means unsupervised clustering of the SOM.

By superimposing the classification information, the map grid resulting by the training of the SOM is as in Figure 6, where each hexagon reports the classification label of documents sharing a given BMU (within brackets, the number of associated documents). From Figure 6, it can be observed that the unsupervised clustering produced by the SOM algorithm is quite coherent with the actual classification given by process operators; in fact ECR documents sharing the same classification label are included in BMUs belonging to the same cluster. It is worth noting that the actual label is assigned to each document after the SOM unsupervised training has been carried out. From Figure 6, it can be also noted that ECRs, classified either as “PS” and “HP,” are all included in a unique cluster. Furthermore, two documents out of twelve with label “MS” are assigned to clusters that include different labels, namely, “CR,” “PS,” and “HP.” We investigated this misclassification and we found that causes of changes described in these two “MS” documents are quite similar to those contained in documents labeled as “CR,” “PS,” and “HP.”

Given the actual classification, the quality of the obtained clustering can be evaluated by computing four indices: purity, precision, recall, and *F*-measure [36].

Let *𝒯* = {*𝒯*_1_,…, *𝒯*_*r*_,…, *𝒯*_*R*_} be the true partitioning given by the process operators, where the partition *𝒯*_*r*_ consists of all the documents with label *r*. Let *m*_*r*_ = |*𝒯*_*r*_| denote the number of documents in true partition *𝒯*_*r*_. Also let *𝒞* = *𝒞*_1_,…, *𝒞*_*k*_,…, *𝒞*_*K*_ denote the clustering obtained via the SOM text clustering algorithm, and *n*_*k*_ = |*𝒞*_*k*_| denote the number of documents in cluster *𝒞*_*k*_. The *K* × *R* contingency matrix **N** induced by clustering *𝒞* and the true partitioning *𝒯* can be obtained by computing the elements **N**(*k*, *r*) = *n*_*kr*_ = |*𝒞*_*k*_∩*𝒯*_*r*_|, where *n*_*kr*_ denotes the number of documents that are common to cluster *𝒞*_*k*_ and true partition *𝒯*_*r*_. The contingency matrix for SOM text clustering of ECRs is reported in Table 2.

Starting from Table 2, the following indices are computed:

(i) *Purity Index*. The cluster-specific purity index of cluster *𝒞*_*k*_ is defined as
  (8) $$\begin{array}{c} {purity}_{k}=\frac{1}{n_{k}}\overset{R}{\underset{r=1}{max}}\left\{\;n_{kr}\;\right\}. \end{array}$$
 The overall purity index of the entire clustering *𝒞* is computed as
  (9) $$\begin{array}{c} purity=\overset{K}{\underset{k=1}{\sum }}\frac{n_{k}}{N}{purity}_{k}=\frac{1}{N}\overset{K}{\underset{k=1}{\sum }}\overset{R}{\underset{r=1}{max}}\left\{\;n_{kr}\;\right\}. \end{array}$$
 As shown in Table 3, clusters *𝒞*_1_, *𝒞*_4_, and *𝒞*_5_ have purity index equal to 1; that is, they contain entities from only one partition. Clusters *𝒞*_2_ and *𝒞*_3_ gather entities from different partitions, that is, *𝒯*_1_, *𝒯*_5_, and *𝒯*_6_ for cluster *𝒞*_2_ and *𝒯*_1_, *𝒯*_2_ for cluster *𝒞*_3_. The overall purity index is equal to 0.79.
(ii) *Precision Index*. Given a cluster *𝒞*_*k*_, let *𝒯*_*r*_*k*__ denote the majority partition that contains the maximum number of documents from *𝒞*_*k*_; that is, *r*_*k*_ = argmax_*r*=1_^*R*^⁡*n*_*kr*_. The* precision* index of a cluster *𝒞*_*k*_ is given by
  (10) $$\begin{array}{c} prec_{k}=\frac{1}{n_{k}}\overset{R}{\underset{r=1}{max}}\left\{\;n_{kr}\;\right\}=\frac{n_{kr_{k}}}{n_{k}}. \end{array}$$
 For clustering in Table 2 the majority partitions are *𝒯*_3_1__, *𝒯*_6_2__, *𝒯*_2_3__, *𝒯*_4_4__, and *𝒯*_1_5__. Precision indices show that all documents gathered in clusters *𝒞*_1_, *𝒞*_4_, and *𝒞*_5_ belong to the corresponding majority partitions *𝒯*_3_1__, *𝒯*_4_4__, and *𝒯*_1_5__. For cluster *𝒞*_2_ the 50% of documents belong to *𝒯*_6_2__ and finally the 88% of documents in cluster *𝒞*_3_ belong to *𝒯*_2_3__.
(iii) *Recall Index*. Given a cluster *𝒞*_*k*_, it is defined as
  (11) $$\begin{array}{c} recall_{k}=\frac{n_{kr_{k}}}{\left|T_{r_{k}}\right|}=\frac{n_{kr_{k}}}{m_{r_{k}}}, \end{array}$$
 where *m*_*r*_*k*__ = |*𝒯*_*r*_*k*__|. It measures the fraction of documents in partition *𝒯*_*r*_*k*__ shared in common with cluster *𝒞*_*k*_. The recall indices reported in Table 3 show that clusters *𝒞*_1_, *𝒞*_2_, *𝒞*_3_, and *𝒞*_4_ shared in common the 100% of documents in majority partitions *𝒯*_3_1__, *𝒯*_6_2__, *𝒯*_2_3__, and *𝒯*_4_4__, respectively. Cluster *𝒞*_5_ shared the 83% of documents in *𝒯*_1_5__.
(iv) *F*-*Measure Index*. It is the harmonic mean of the precision and recall values for each cluster. The *F*-measure for cluster *C*_*k*_ is therefore given as
  (12) $$\begin{array}{c} F_{k}=\frac{2\cdot prec_{k}\cdot recall_{k}}{prec_{k}+recall_{k}}=\frac{2n_{kr_{k}}}{n_{k}+m_{r_{k}}} \end{array}$$
 The overall *F*-measure for the clustering *𝒞* is the mean of the clusterwise *F*-measure values:
  (13) $$\begin{array}{c} F=\frac{1}{K}\overset{K}{\underset{k=1}{\sum }}F_{k}. \end{array}$$
 
Table 3 shows that *F*-measure of clusters *𝒞*_1_ and *𝒞*_4_ is equal to 1, while other values are less than 1. The low values of *F*-measures for clusters *𝒞*_2_, *𝒞*_3_, and *𝒞*_5_ depend on a low precision index for clusters *𝒞*_2_ and *𝒞*_3_ and on a low recall index for cluster *𝒞*_5_. Consequently, the overall *F*-measure is equal to 0.90.

Given the actual classification, the SOM can be further validated through a leave-one-out cross validation technique in order to check its classification ability. In particular, *N* − 1 ECR documents are used for training and the remaining one for testing (iterating until each ECR text in the data has been used for testing).

At each iteration, once SOM has been trained on *N* − 1 ECRs, and when the testing sample is presented as input, a BMU is selected in the matching step of the SOM algorithm. The label of the training documents associated with that BMU is considered. In the case of an empty BMU, that is, which results are not associated with any training documents, the closest one associated with at least one training document is considered instead, while in case of a BMU associated with training documents with more than one label, the label with greater number of documents is considered.

Table 4 shows the results of the leave-one-out cross validation. For each row, that is, for a given ECR label, the second column reports the total number of documents in the dataset, while the last two columns report the number of testing ECRs correctly classified by the SOM. In particular, the third column reports the number of testing ECRs correctly classified as they were connected to a first BMU associated with training documents with the same label. The last column refers to the number of ECRs associated with an empty first BMU that, nevertheless, resulted closest to a second BMU related to documents belonging to the same class of the testing sample. Also cross validation study demonstrates that labels given by SOM are coherent with actual classification and confirms the ability of SOM as classification tool.

## 6. Conclusions

In this paper, a real case study regarding the engineering change process in complex products industry was conducted. The study concerned the postchange stage of the engineering change process, in which past engineering changes data are analyzed to discover information exploitable in new engineering changes. In particular, SOM was used for clustering of natural language written texts produced during the engineering change process. The analyzed texts included the descriptions of the causes of changes contained in the ECR forms. Firstly, SOM algorithm was used as clustering tool to find relationships between the ECR texts and to cluster them accordingly. Subsequently, SOM was tested as classification tool and the results were validated through a leave-one-out cross validation technique.

The results of the real case study showed that the use of the SOM text clustering can be an effective tool in improvement of the engineering change process analysis. In particular, some of the advantages highlighted in this study are as follows:

1. Text mining methods allow analyzing unstructured data and deriving high-quality information. The main difficulty in ECR analysis consisted in analyzing natural language written texts.
2. Clustering analysis of past ECRs stored in the company allows automatically gathering ECRs on the basis of similarity between documents. When a new change triggers, the company can quickly focus on the cluster of interest. Clustering can support the company to know if a similar change was already managed in the past, to analyze the best solution adopted and to learn avoiding the same mistakes made in the past.
3. Use of SOM for ECRs text clustering allows automatically organizing large documents collection. With respect to other clustering algorithms, the main advantage of SOM text clustering is that the similarity of the texts is preserved in the spatial organization of the neurons. The distance among prototypes in the SOM map can therefore be considered as an estimate of the similarity between documents belonging to clusters. In addition, SOM can first be computed using a representative subset of old input data. New input can be mapped straight into the most similar model without recomputing the whole mapping.

Nevertheless, the study showed some limitations of the application of SOM text clustering and classification. A first limitation is linked to natural language written texts. The terms contained in different texts may be similar even if an engineering change request concerns a different product. The similarity of terms may influence the performance of SOM-based clustering. A second limitation is linked to the use of SOM as classification method. Classification, indeed, requires the labeling of a training dataset. This activity requires a deep knowledge of the different kinds of ECRs managed during the engineering change process and may be difficult and time consuming.

As a summary, research on use of SOM text clustering in engineering change process analysis appears to be a promising direction for further research. A future direction of the work will consider the use of SOM text clustering on a larger dataset of ECRs comparing SOM with other clustering algorithms such as *K*-means or hierarchical clustering methods. Another direction of future research concerns the analysis of SOM robustness to parameters selection (i.e., the size and structure of the map, parameters and kinds of learning, and neighborhood functions).

## Figures and Tables

**Table 1:** Class, label, and number of ECR texts.

Class	Label	Number of ECR texts
(1) Metal-Sheet	MS	12
(2) Carter	CR	8
(3) Antenna	AN	8
(4) Semi-Finished Round	SR	7
(5) Hydraulic Panel	HP	9
(6) Pneumatic System	PS	10

**Table 2:** Contingency matrix **N**.

	MS	CR	AN	SR	HP	PS	*n*_*r*_
	*𝒯*_1_	*𝒯*_2_	*𝒯*_3_	*𝒯*_4_	*𝒯*_5_	*𝒯*_6_	
*C*_1_	0	0	8	0	0	0	8
*C*_2_	1	0	0	0	9	10	20
*C*_3_	1	8	0	0	0	0	9
*C*_4_	0	0	0	7	0	0	7
*C*_5_	10	0	0	0	0	0	10
*m*_*k*_	12	8	8	7	9	10	*N* = 54

**Table 3:** Clustering validation.

Cluster-specific and overall indices				
Purity				

purity_1_ = 1	purity_2_ = 0.5	purity_3_ = 0.89	purity_4_ = 1	purity_5_ = 1
purity = 0.79				

Precision				

prec_1_ = 1	prec_2_ = 0.5	prec_3_ = 0.89	prec_4_ = 1	prec_5_ = 1

Recall				

recall_1_ = 1	recall_2_ = 1	recall_3_ = 1	recall_4_ = 1	recall_5_ = 0.83

*F-*measure				

*F*_1_ = 1	*F*_2_ = 0.66	*F*_3_ = 0.94	*F*_4_ = 1	*F*_5_ = 0.91
*F* = 0.90				

**Table 4:** Results of SOM-based classification. In the first and in the second column, labels and number of ECR texts of the actual classification are reported. In the third column, the number of ECRs correctly classified through the label of first associated BMU. In the fourth column, the number of document associated with an empty first BMU but correctly classified by considering the label of documents sharing the second associated BMU.

Labels	Number of ECRs	Number of ECRs classified through the 1st BMU	Number of ECRs classified through the 2nd BMU
MS	12	10	2
CR	8	6	2
AN	8	7	1
SR	7	6	1
HP	9	6	3
PS	10	10	—

## Symbols

*α*(*t*): Scalar-valued learning-rate factor
**N**: Contingency matrix
*δ*(*𝒞*_*k*_, *𝒞*_*l*_): Between-cluster distance
Δ(*𝒞*_*k*_): Within-cluster distance
Δ**m**_*i*_(*t*): Adjustment of the prototype vector **m**_*i*_(*t*)
*𝒞*: Collection of documents
*𝒞*_*k*_: Cluster of documents of index *k*
*𝒯*: True partition of documents given by process operators
*𝒯*_*r*_: True partition of documents with label *r*
*𝒯*_*r*_*k*__: Majority partition containing the maximum number of documents from *𝒞*_*k*_
*σ*(*t*): Width of the kernel which corresponds to the radius of *N*_*c*_(*t*)
*c*(**x**_*j*_): Index of the BMU associated with **x**_*j*_
*d*_*j*_: Document of index *j*
DBI(*K*): Davies Bouldin Index for *K* clusters
*df*_*p*_: Number of documents in the collection *𝒞* which contains term *p*
*h*_*ci*_(*t*): Neighborhood function
*K*: Total number of clusters
*K*^*∗*^: Optimum number of clusters
*M*: Total number of terms in the documents collection
*m*_*r*_: Number of documents in partition *𝒯*_*r*_
*N*: Total number of processed documents
*N*_*c*_(*t*): Neighborhood around node *c*
*n*_*k*_: Number of documents in cluster *𝒞*_*k*_
*n*_*kr*_: Number of documents common to cluster *𝒞*_*k*_ and partition *𝒯*_*r*_
*P*: Total number of units of SOM
*t*: Index of time
*tf*_*p*,*j*_: Term frequency of term *p* in the *j*th document
*w*_*p*,*j*_: Weight associated with term *p* in the *j*th document
**m**_*c*_: Prototype vector associated with the BMU
**m**_*i*_: Prototype vector of index *i*
**r**_*c*_: Position vector of index *c*
**r**_*i*_: Position vector of index *i*
**x**_*j*_: Numerical feature vector associated with *j*th document.
